# 1233. Leveraging Informatics to Evaluate and Sustain Improvements in Peri-Operative Antimicrobial Prophylaxis Achieved Under the Surgical Care Improvement Project (SCIP)

**DOI:** 10.1093/ofid/ofad500.1073

**Published:** 2023-11-27

**Authors:** Westyn Branch-Elliman, Samuel Golenbock, Kierstin Hederstedt, Jacquelyn Pendergast, Marlena Shin, Ryann Engle, A Rani Elwy, Katie Colborn, Mary Hawn, Hillary J Mull

**Affiliations:** VA Boston Healthcare System, West Roxbury, MA; VA Boston Healthcare System, West Roxbury, MA; VA Boston CHOIR, Boston, Massachusetts; VA Boston CHOIR, Boston, Massachusetts; VA Boston Healthcare System, West Roxbury, MA; VA Boston CHOIR, Boston, Massachusetts; VA Bedford Healthcare System, Bedford, Massachusetts; University of Colorado, Denver, Colorado; Stanford University, Palo Alto, California; VA Boston Healthcare System, West Roxbury, MA

## Abstract

**Background:**

The Surgical Care Improvement Project (SCIP) was a national quality improvement program designed to improve peri-operative outcomes. The SCIP Program included several guideline-based antimicrobial stewardship measures, including discontinuation of antibiotics within 48 hours of skin closure for cardiac surgeries (INF-3). INF-3 was retired at the end of 2015; since this time, no systematic tools have been developed to support ongoing quality assessments.

**Methods:**

We developed a retrospective, national cohort of cardiac surgeries in the national VA healthcare system during the period from 2010-2015 and merged these data with data from the VA External Peer Review Program, which included manually-assessed SCIP metrics, including INF-3. We electronically re-created the SCIP program by mapping exclusion criteria and developing code to assess the duration of post-operative antimicrobial use (**Table 1**). Among the manually-adjudicated cohort, we then iteratively refined the electronic tool until pre-specified criterion validity were achieved. After development, INF-3 compliance as assessed manually and by the objective informatics tool were compared.

**Figure:**
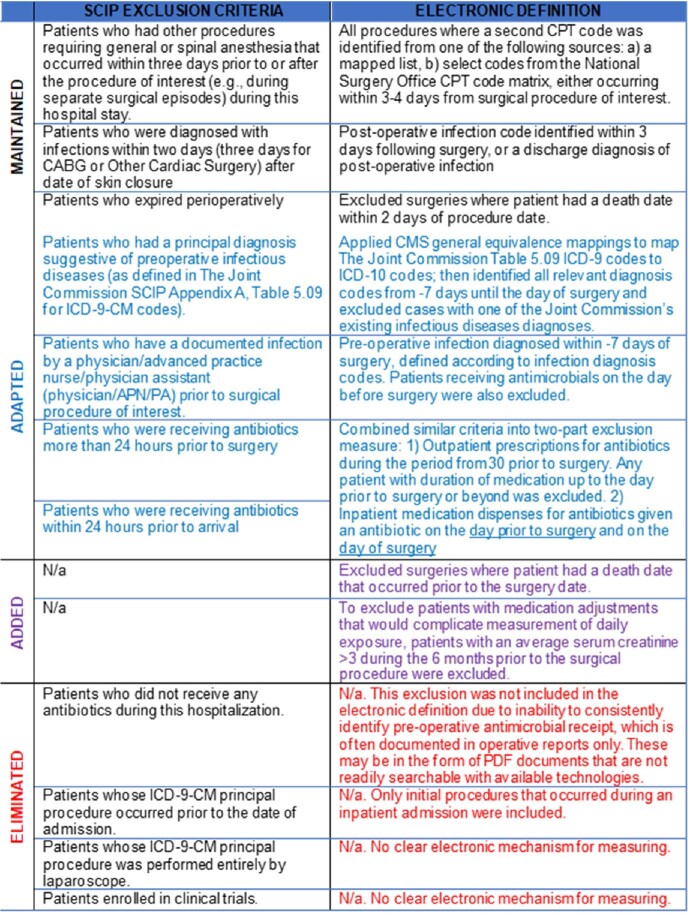

**Results:**

During the study period, 11,361 cardiac surgeries representing 26 VA facilities met inclusion criteria. The overall estimated compliance rate was 97.7% in the EPRP-reviewed cases (N=11,361) and 95.9% as-assessed by the informatics tool (N=9,561 cases); facility quality rankings using both methods were similar but not exactly the same (**Figure 1**). Facility-level compliance trends were similar using both measurement methodologies and the correlation between the two measures was high (r=0.69); however, the informatics tool consistently estimated compliance to be approximately 1.9% lower than manually reviewed cases (**Figure 2**). As estimated by the manual review program, 16 facilities (61.5%) achieved >97% compliance rates versus 9 facilities (34.6%) as estimated by the informatics tool.

**Figure 2.** Facility-Level Compliance with INF-3 as Measured by Manual Review versus via Objective Electronic Tool

**Figure:**
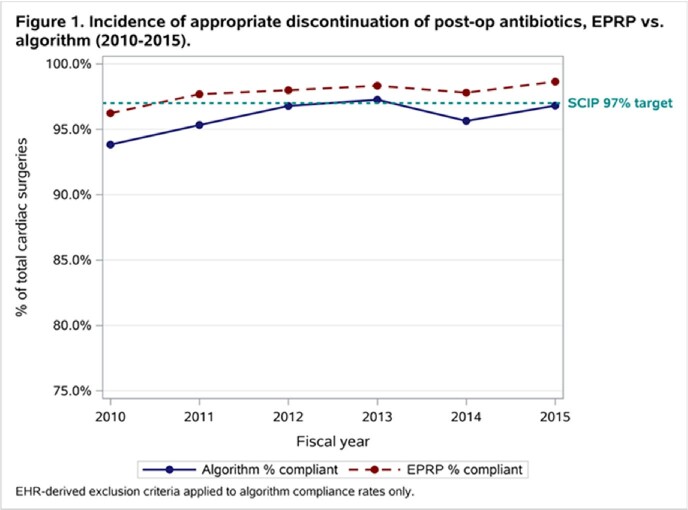

**Figure:**
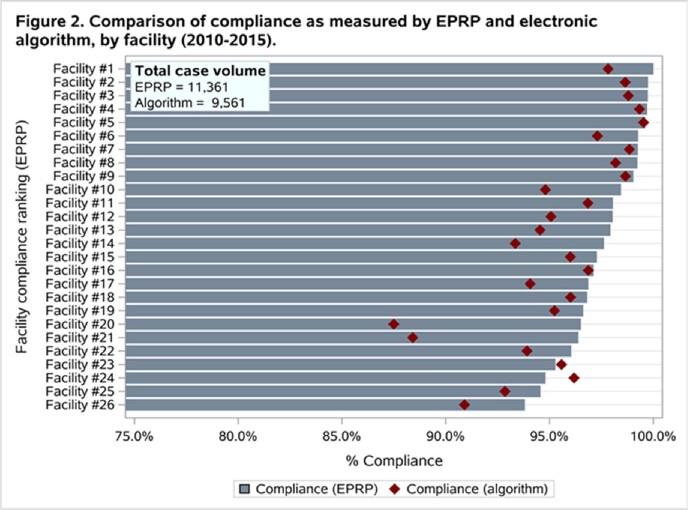

**Conclusion:**

We developed a comprehensive, objective informatics tool for assessing ongoing compliance with SCIP INF-3. The tool can be applied in future investigations to assess the sustainability of practice changes achieved under the SCIP program and to identify areas for future improvement.

**Disclosures:**

**Westyn Branch-Elliman, MD, MMsc**, DLA Piper, LLC/Medtronic: Advisor/Consultant|DLA Piper, LLC/Medtronic: Expert Testimony|Gilead Sciences: Grant/Research Support

